# Cathelicidins in the Tasmanian devil (*Sarcophilus harrisii*)

**DOI:** 10.1038/srep35019

**Published:** 2016-10-11

**Authors:** E. Peel, Y. Cheng, J. T. Djordjevic, S. Fox, T. C. Sorrell, K. Belov

**Affiliations:** 1 Faculty of Veterinary Science, The University of Sydney, Sydney, Australia; 2 Centre for Infectious Diseases and Microbiology, The Westmead Institute for Medical Research, and Marie Bashir Institute for Infectious Diseases and Biosecurity, University of Sydney, Sydney, Australia; 3 Department of Primary Industries, Parks, Water and Environment, 134 Macquarie Street, Hobart, Tasmania 7000, Australia

## Abstract

Tasmanian devil joeys, like other marsupials, are born at a very early stage of development, prior to the development of their adaptive immune system, yet survive in a pathogen-laden pouch and burrow. Antimicrobial peptides, called cathelicidins, which provide innate immune protection during early life, are expressed in the pouch lining, skin and milk of devil dams. These peptides are active against pathogens identified in the pouch microbiome. Of the six characterised cathelicidins, Saha-CATH5 and 6 have broad-spectrum antibacterial activity and are capable of killing problematic human pathogens including methicillin-resistant *S. aureus* and vancomycin-resistant *E. faecalis*, while Saha-CATH3 is active against fungi. Saha-CATH5 and 6 were toxic to human A549 cells at 500 μg/mL, which is over seven times the concentration required to kill pathogens. The remaining devil cathelicidins were not active against tested bacterial or fungal strains, but are widely expressed throughout the body, such as in immune tissues, in digestive, respiratory and reproductive tracts, and in the milk and pouch, which indicates that they are likely also important components of the devil immune system. Our results suggest cathelicidins play a role in protecting naive young during pouch life by passive immune transfer in the milk and may modulate pouch microbe populations to reduce potential pathogens.

Antimicrobial peptides are a primitive component of the innate immune system of animals, with cathelicidins and defensins being the two predominant families in mammalian species^1^. Cathelicidins are a family of small, cationic antimicrobial peptides, which function as natural antibiotics through antimicrobial and immunomodulatory activities^1^. Cathelicidins directly kill a broad spectrum of bacteria^2^, fungi^3^ and parasites^4^ through electrostatic interaction of positively charged peptides with the negative pathogen cell membrane, resulting in the formation of trans-membrane pores^5^. In addition, cathelicidins indirectly target pathogens by modulating cells of the innate and adaptive immune systems to alter the local cellular and chemical environment^1^. This includes; the release and suppression of pro-inflammatory mediators^6^,^7^,^8^, formation of chemotactic gradients^9^, immune cell development and angiogenesis^10^,^11^. Cathelicidins are encoded as prepropeptides which are stored within neutrophil granules and epithelial cells. The prepropeptides contain three domains; a signal peptide and conserved cathelin domain provides structure to the peptide through the formation of two di-sulphide bonds between four cysteine residues^12^. The antimicrobial domain is positively charged and varies significantly within and amongst individual species. Following secretion, the antimicrobial domain is cleaved to form the active mature peptide^1^.

Cathelicidins have been studied extensively in eutherian mammals, however marsupials are relatively unexplored. Marsupials have a characteristic short gestation period of up to only 35 days^13^ culminating in the birth of highly altricial young^14^,^15^. Young are born immunologically naive due to undifferentiated lymphoid tissues and lack of immune effector cells^16^. Unlike eutherians, which undergo immune development within the sterile confines of the uterus, marsupials undergo this process within a pouch or burrow that contains a diverse community of bacteria and fungi^17^. Primary immune tissues do not mature for up to 30 days after birth^16^ and antibody-mediated immunity is not fully acquired until around 90 days^18^. Mechanisms which protect the young during this time are not fully understood, but rapid development of the innate immune system and passive transfer of antibodies and antimicrobial peptides in the milk are two strategies thought to be involved^19^. Cathelicidins are secreted in tammar wallaby milk throughout lactation^20^ and expressed in the pouch epithelium^14^,^21^,^22^ and pouch young skin from day one post-partum^22^. At these sites they may modulate microbial populations to eliminate pathogens^23^, supporting their functional role in protecting naive young.

Evolution of the cathelicidin gene family through gene duplications has resulted in multiple genes in different lineages, encoding separate peptides^24^. The need to protect immunologically naive young may have encouraged the lineage specific expansion of the cathelicidin gene family in marsupials^14^. Marsupials have a higher number of cathelicidin genes than eutherians. For example, the gray short-tailed opossum (*Monodelphis domestica*) has 12 cathelicidin genes, whereas humans^25^ and mice^26^ have only one. Cathelicidins have only been characterised in the tammar wallaby (*Macropus eugenii*), gray short-tailed opossum and platypus (*Ornithorhyncus anatinus*) due to the availability of genomic data. Only two tammar wallaby cathelicidins have been tested, and both show broad spectrum antimicrobial activity and are capable of killing antibiotic resistant *Pseudomonas aeruginosa* and *Klebsiella pneumoniae*^22^. These results suggest marsupial cathelicidins may provide an alternative to traditional antibiotics in the fight against drug resistant pathogens. Given the range of activity observed across tammar wallaby and eutherian cathelicidins, we wanted to explore the antimicrobial activity of other marsupial cathelicidins and their potential role in protecting pouch young. Tasmanian devils have a short gestation period of up to 30 days and give birth to young weighing only 0.3 g^27^. Young remain firmly attached to the teat for the next 80 days^27^, after which they ride on the mother’s back and are left in a den, but continue to suckle until weaning at around 7 months^28^. In addition to the need for protection during pouch life, Tasmanian devils are exposed to a range of harmful pathogens during adulthood. Tasmanian devils are scavengers and are renowned for their aggressive behaviour, including fighting and biting before they become independent adults^28^. As such, their immune system may have evolved mechanisms such as cathelicidins with broad-spectrum antimicrobial activity to compensate for this heightened exposure. Release of the Tasmanian devil genome in 2012 provided a new source of data to search for cathelicidins^29^. Our aim was to characterise cathelicidins in the Tasmanian devil genome, and determine their antimicrobial activity against human and animal pathogens with the aim of understanding their role in protecting Tasmanian devil young during pouch life. Additional findings may lead to possible antimicrobial candidates for future therapeutic development.

## Results

### Cathelicidin gene characterization

Six cathelicidins were identified in the Tasmanian devil genome. Cathelicidin names were assigned at random and do not imply their position on the chromosome or orthology with other species. All six cathelicidins are located on chromosome two^29^, however their relative positions on the chromosome are unknown due to the fragmented nature of the genome assembly.

Multiple sequence alignment revealed that Tasmanian devil peptides contained all the characteristic features of cathelicidins, including peptide domains and motifs (Supplementary Figure S1). All six genes contained four exons, similar to other cathelicidins. The prepropeptides ranged from 154 to 172 amino acids in length and contained a signal peptide, conserved cathelin domain and variable antimicrobial domain.

The signal peptide sequence ranged from 21–27 amino acids in length with a high proportion of leucine residues. The cathelin domain ranged from 89 to 95 amino acid residues in length and contained a number of conserved residues. The characteristic four cysteine motif, which is an identifying feature of cathelicidins, was present and is shown in Supplementary Figure S1. The antimicrobial domain was highly variable in length and composition, ranging from 20 to 37 amino acids with a high proportion of charged residues. This imparts a cationic charge on the mature peptide, ranging from 3.9 to 8.1 at pH7 (Table 1). Few conserved residues were evident, with only 17 to 47% similarity amongst Tasmanian devil mature peptide sequences. Significant sequence heterogeneity was also observed within marsupial mature peptides, as Tasmanian devil sequences were only 15–40% similar to tammar wallaby and 3–30% to opossum.

**Figure 1:**
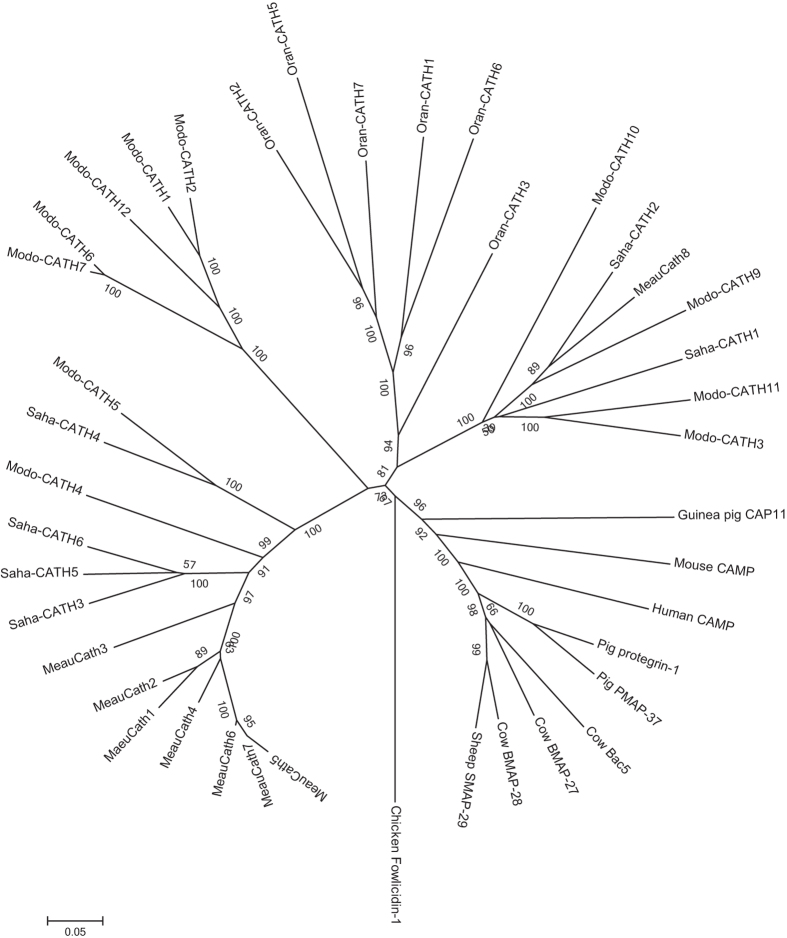
Neighbour joining phylogenetic tree showing the evolutionary relationships among Tasmanian devil Saha-CATH1 to 6, tammar wallaby MaeuCath 1 to 8, opossum Modo-CATH1 to 7 and 9 to 12, platypus Oran-CATH1 to 7, human CAMP, mouse CAMP, pig Protegrin-1 and PMAP-37, cow Bac5, BMAP-27 and BMAP-28, sheep SMAP-29 and chicken fowlicidin-1.

As expected, Tasmanian devil cathelicidins cluster with those of other marsupials in the phylogenetic tree (Fig. 1), indicating that they are more similar to marsupial cathelicidins than to those of monotremes or eutherians. Saha-CATH1 and 2 cluster with opossum and tammar wallaby cathelicidins, suggesting that these genes arose prior to the divergence of these species around 70 million years ago^30^. In comparison, Saha-CATH3, 5 and 6 form a species-specific clade, which suggests that they have arisen through more recent gene duplications.

### Expression profile

**Figure 2:**
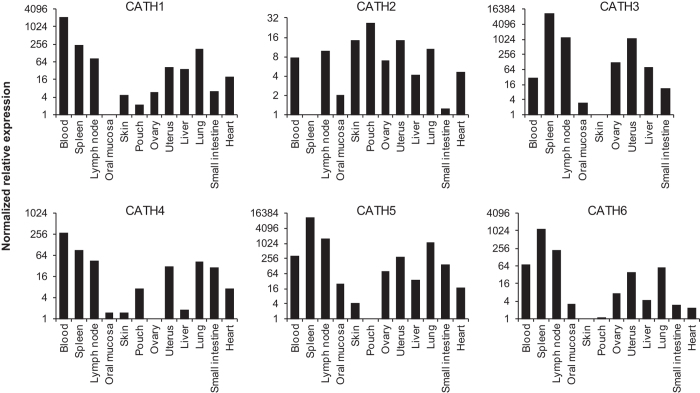
Relative fold expression of Tasmanian devil cathelicidin genes in different tissues. For each cathelicidin, relative expression was calculated in comparison to the tissue with the lowest expression (i.e. the tissue with fold 1 expression in each graph). Three tissues (pouch, lung, and heart) that showed no detectable Saha-CATH3 expression were not included in the CATH3 graph.

Expression level of Tasmanian devil cathelicidins were assessed in a wide range of immune, reproductive, secretory, respiratory and gastrointestinal tissues using relative qPCR (Fig. 2). Saha-CATH1, 2, 4, 5 and 6 were expressed in all tissues tested, whilst Saha-CATH3 was not expressed in the pouch, lung or heart. As expected, all six cathelicidins were present in blood, spleen and lymph node as they are stored within neutrophil granules. Saha-CATH1 and 4 were most highly expressed in the blood, whilst Saha-CATH3, 5 and 6 were most highly expressed in the spleen. Conversely, Saha-CATH2 was present at the lowest levels within the spleen and was most highly expressed in the pouch. Saha-CATH1, 4, 5 and 6 were also expressed in the pouch at lower levels compared to their expression in other tissues. Additionally, transcripts of Saha-CATH1, 2, 5 and 6 were also detected in a milk transcriptome^31^. Cathelicidin expression within the mouth mucosa, skin, pouch, uterus and milk supports their functional role in protecting naïve young during development.

### Change of pouch microbiome during lactation

**Figure 3:**
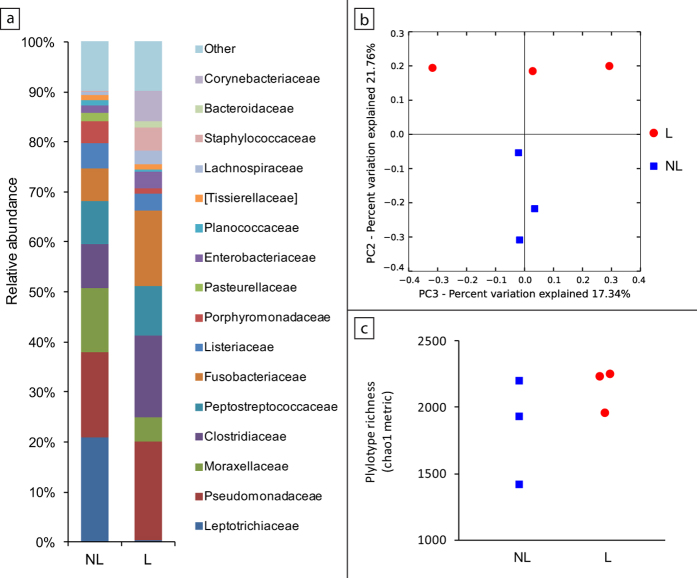
Comparison of pouch microbiome between non-lactating (NL) and lactating (L) Tasmanian devils. (**A**) Relative abundance of bacterial families. (**B**) PCoA analysis of unweighted UniFrac distances. (**C**) Bacterial phylotype richness inferred using Chao1 metric and 40,000 sequences for rarefaction.

To test the hypothesis that the microbial community in devil’s pouch undergoes compositional changes in response to lactation, we examined the pouch microbiota of three non-lactating and three lactating devils by sequencing PCR amplicons of the bacterial 16S rRNA gene V3-V4 region (around 465 bp) on the Illumina MiSeq System. A total of 468,268 sequences were obtained after quality filtering, with the smallest number of sequences obtained for one sample being 44,880 and the largest being 116,364. These sequences were grouped into 4,410 operational taxonomic units (OTUs), which were categorised to 26 bacterial phyla, 79 classes, 138 orders, 261 families, and 487 genera. Consistent with previous observation^32^, the pouch flora of non-lactating devils consisted mainly of bacteria from five phyla: Proteobacteria (35.3%), Firmicutes (28.4%), Fusobacteria (27.5%), Bacteroidetes (5.7%), and Actinobacteria (1.9%) (Fig. 3).

Several differences were detected between the pouch of non-lactating and lactating devils (Fig. 3). Firstly, the unweighted UniFrac distance within the non-lactating group (0.58 ± 0.03) was significantly lower than the distance between lactating and non-lactating samples (0.68 ± 0.07), suggesting a high degree of compositional dissimilarity between the pouch flora of non-lactating and lactating devils. This can be seen in the principal coordinates analysis (PCoA) plot (Fig. 3) where non-lactating samples grouped together but were separate from the lactating samples. Thirty-four OTUs exhibited significantly lower relative abundance in the lactating pouch than in non-lactating samples (Wilcoxon rank sum test p < 0.05), with the most pronounced changes occurring in the level of Leptotrichiaceae (20.9% in non-lactating samples vs. 0.4% in lactating), Porphyromonas (4.5% vs. 0.3%), Pasteurellaceae (1.7% vs. 0.1%), and Parvimonas (1.0% vs. 0.2%). Despite the decrease in these bacteria, the overall level of bacterial diversity in the pouch of lactating and non-lactating devils was similar (Fig. 3), which may be a result of faecal contamination from the pouch young. Indeed, the prevalence of Cetobacterium (1.9% in non-lactating samples vs. 10.9% in lactating) and Clostridium (1.0% vs. 4.3%), two genera that are main components of devil gut microbiota, significantly increased in the pouch that had pouch young present.

### Antimicrobial activity

To assess the antimicrobial activity of devil cathelicidins, we tested synthesized mature peptides on 25 bacterial and 6 fungal strains (Table 2). As there were no Tasmanian devil specific isolates available, human and veterinary strains were used. The antimicrobial activity of Tasmanian devil cathelicidin mature peptides Saha-CATH1 to 6 against bacteria and fungi is summarised in Table 2. Saha-CATH1, 2 and 4 did not show antimicrobial activity against the bacteria and fungi tested, with a minimum inhibitory concentration (MIC) of greater than 64 μg/mL. As such, these values are not included in Table 2. Saha-CATH3 had highly specific activity, as it was unable to kill any bacteria, but killed *Cryptococcus neoformans*. Saha-CATH6 killed a number of *Streptococcus* species and vancomycin-resistant *Enterococcus faecalis* (VREF), however was ineffective against other bacteria. On the other hand, Saha-CATH5 had broad-spectrum antibacterial activity against gram-negative and gram-positive bacteria and killed the drug resistant pathogens VREF and methicillin-resistant *Staphylococcus aureus* (MRSA) (Table 2). The antifungal activity of Saha-CATH5 and 6 was more restricted, but both peptides showed activity against *Candida krusei*, *Cryptococcus neoformans* and *Cryptococcus gattii*. The MIC values for ampicillin, tetracycline and fluconazole were within the acceptable limits for ATCC strains according to clinical and laboratory standards institute (CLSI) guidelines, and are included in Table 2.

### Cytotoxicity and haemolysis

**Figure 4:**
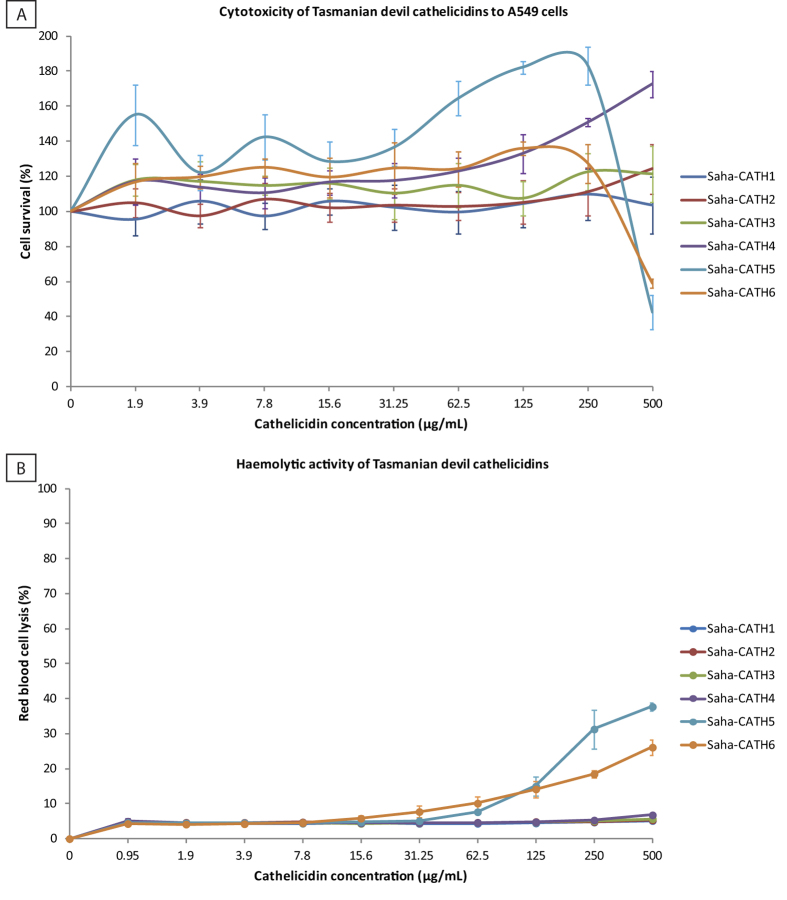
Toxicity of Tasmanian devil cathelicidins Saha**-**CATH1 to 6 to human cells. (**a**) cytotoxic activity against the human A549 cell line expressed as percent cell survival compared to untreated control cells. (**b**) human haemolytic activity expressed as percent red blood cell lysis compared to the 1% Triton X-100 positive control. The mean values ± SD (bars) of two independent experiments performed in duplicate are reported.

Further experiments were carried out to assess cytotoxic and haemolytic potencies of devil cathelicidin mature peptides. Tasmanian devil cathelicidin mature peptides Saha-CATH1, 2, 3 and 4 were not toxic to the human cell line A549 as seen in Fig. 4a. Cell survival of Saha-CATH1 treated cells remained close to the control at all concentrations. Survival of Saha-CATH2 treated cells also remained close to the control over dilutions ranging between 1.9 μg/mL to 250 μg/mL, however survival of cells at 500 μg/mL was significantly higher than the growth control (p < 0.05). The same pattern was observed for Saha-CATH3 at 1.9, 7.9, 15.6 and 250 μg/mL and all dilutions of Saha-CATH4 ranging between 15.6 to 500 μg/mL (p < 0.05). The significant increase in cell survival above the level of the untreated growth control, as seen in Fig. 4a, does not necessarily indicate cathelicidins are inducing proliferation of A549 cells, rather it is more likely a reflection of cell growth throughout the assay. Alamar blue is an indicator of mitochondrial activity, hence cell growth during the assay results in an increase in the reduction of alamar blue and an elevated percentage cell survival relative to the growth control. Cell survival of Saha-CATH5 and 6 treated cells was significantly higher than the growth control over the concentrations of 1.9 to 250 μg/mL, and 3.9 to 250 μg/mL respectively (p < 0.05). As such, Saha-CATH5 and 6 were non toxic up to a concentration of 250 μg/mL. Saha-CATH5 was the most toxic as treatment of cells with 500 μg/mL resulted in 42% cell survival compared to the untreated growth control (p < 0.05). Similarly, treatment with Saha-CATH6 at the same concentration resulted in 59% cell survival (p < 0.05).

The majority of Tasmanian devil cathelicidin mature peptides did not lyse human red blood cells as shown in Fig. 4b. Saha-CATH1, 2, 3 and 4 caused very low levels of haemolysis, below 7% at all concentrations compared with the positive control. Despite this, mean absorbance values for red blood cells treated with Saha-CATH2 at 500 μg/mL, Saha-CATH3 at 250 and 500 μg/mL and Saha-CATH4 from 125 to 500 μg/mL were significantly different from the negative control (p < 0.05). Similarly, mean absorbance values of Saha-CATH5 and 6 from 31.25 to 125 μg/mL were significantly different from the negative control (p < 0.05), however lysis remained below 15% at these concentrations. Saha-CATH6 was moderately haemolytic at 250 and 500 μg/mL, however lysis remained below 26% (p < 0.05). In support of the cytotoxicity data above, Saha-CATH5 was the most toxic as it caused 37% haemolysis at 500 μg/mL (p < 0.05).

## Discussion

Tasmanian devils have multiple cathelicidin genes, similar to other marsupials^14^,^22^,^33^, which have evolved to protect immunologically naive young through modulating the pouch microbiome and providing a direct source of immune compounds within the milk. This is supported by the presence of Tasmanian devil cathelicidins Saha-CATH1, 2, 4, 5 and 6 in the milk and pouch, coupled with their antimicrobial activity. Saha-CATH5 and 6 killed bacteria identified in the pouch microbiome as well as drug resistant strains. Saha-CATH3 exhibited activity uniquely against the fungal pathogen, *C. neoformans*. Saha-CATH1, 2 and 4 were not antimicrobial, but the peptides were widely expressed throughout the body and may modulate the immune system.

Our analyses show that Tasmanian devil cathelicidin Saha-CATH5 has broad-spectrum antibacterial activity against gram negative and positive bacteria (Table 2). Tasmanian devil cathelicidin Saha-CATH6 is also antibacterial, however is only active against gram positive strains. Both cathelicidins killed the drug resistant pathogens MRSA and VREF. Fungi which are commonly involved in skin infections (*Candida* spp.) were also killed by Saha-CATH5 and 6, as were *Cryptococcus* species. The activity of Saha-CATH3 was highly selective for *C. neoformans*, which is unusual as cathelicidins generally have broad activity more similar to Saha-CATH5. Furthermore, Saha-CATH3 was not expressed in the lung, a common site of infection for *C. neoformans* in other marsupials such as koalas^34^. Further research is required to identify the mechanism of devil cathelicidin antimicrobial activity, which most likely involves electrostatic interaction and formation of trans-membrane pores similar to eutherian cathelicidins^1^. MIC results presented in Table 2 are based on clinical isolates from humans, livestock and domestic animals as devil isolates were not available. Given this, the activity of devil cathelicidins may differ when tested against devil bacteria due to host-pathogen co-evolution and development of resistance to cathelicidins. The possibility of resistance is an important aspect which warrants further investigation.

The Tasmanian devil pouch contains diverse microbial flora as seen in Fig. 3. Previous studies in the tammar wallaby and quokka have shown that the number of gram negative bacteria such as *E. coli* and *P. aeruginosa*^23^,^35^ decrease in the pouch prior to the birth of young. A significant decrease in the abundance of gram-negative bacteria at the level of Leptotrichiaceae, Porphyromonas and Pasteurellaceae was observed in the pouch of lactating devils, compared to non-lactating. Except for *Pasteurella*, bacteria from these families were not included in our tests and devil cathelicidins were unable to kill *P. multocida*. Saha-CATH5 and 6 are expressed in the pouch and killed *Streptococcus* identified in the pouch microbiome^32^. In addition, Saha-CATH5 and 6 are expressed in the mouth mucosa and may be transferred to the pouch as the mother licks the area. Tasmanian devils and other dasyurid marsupials have been observed grooming the urogenital area and pouch prior to and after birth^36^,^37^.

Interestingly, Saha-CATH3, 5 and 6 cluster together in the phylogenetic tree and are relatively distant from Saha-CATH1, 2 and 4 which did not show antimicrobial activity. This suggests that Saha-CATH3, 5 and 6 are paralogs resulted from more recent duplications in the Tasmanian devil lineage and have undergone lineage-specific diversification. This may have been driven by devils responding to species-specific pathogen pressures and the need to protect naive young through the evolution of highly variable cathelicidins. Tasmanian devil mature peptides are highly variable and share only 40% similarity with tammar wallaby cathelicidins. In contrast, eutherians such as pigs share up to 94% similarity in this domain.

In addition to their antimicrobial role in the pouch and milk, devil cathelicidins expressed in the gut, mouth and skin may be involved in epithelial defence at these sites. The devil gut microbiome contains populations of *Pseudomonas* and *Streptococcus*^32^, both of which are killed by Saha-CATH5 and 6 expressed in the small intestine. Saha-CATH5 and 6 are also expressed in the mouth mucosa and are able to kill *Streptococcus* species identified in the oral microbiome^32^. Similar to Saha-CATH5 and 6, Saha-CATH1, 2 and 4 were expressed in all tissues tested yet did not kill pathogens or human cells. Amongst these peptides Saha-CATH2 has an interesting expression profile, as unlike all other devil cathelicidins, shows lowest levels of expression within the spleen yet is most highly expressed in the pouch. As such, Saha-CATH1, 2 and 4 may play a role in innate immunity in devils. This hypothesis is supported by phylogenetic analysis which shows that Saha-CATH1 and 2 cluster with other tammar wallaby and opossum cathelicidins, in a sister clade to platypus cathelicidins. As these genes have been conserved for over 70 million years of evolution^30^, they must have an essential function in the marsupial and monotreme immune systems. In eutherians, cathelicidins interact with immune cells to activate, suppress and/or enhance the immune system^1^. Cathelicidins form chemotactic gradients to attract immune cells^38^. They also induce and suppress the release of pro-inflammatory mediators^38^,^39^, and are involved in wound healing^40^ and angiogenesis^10^. We propose they have similar activities in marsupials.

Cytotoxicity often hinders the pipeline of progression of peptides to drugs. Non-specific activity against mammalian cells is a common feature of the human cathelicidin LL-37^41^. Cytotoxicity has not been explored in marsupial cathelicidins until now. Tasmanian devil Saha-CATH5 and 6 had non-specific toxicity against a human cell line. While overall mammalian cell membranes are zwitterionic, they contain a large number of negatively charged glycoproteins and glycolipids which could interact with the positively charged peptide^5^. However, the concentrations of Saha-CATH5 and 6 which are toxic to human cells is far higher than that which is required to kill bacteria and fungi. This is most likely because bacterial and fungal cell membranes carry a more negative charge due to the presence of negative lipopolysaccharides or techoic acids^42^.

Tasmanian devil cathelicidins Saha-CATH5 and 6 are potential candidates for drug development. Their broad-spectrum antibacterial activity and ability to kill MRSA and VREF could translate into numerous therapeutic applications. Their non-specific activity against mammalian cells may be reduced or eliminated through identification of active sites within the peptide which are essential for antimicrobial activity and by modification of areas contributing to toxicity. The specificity of Saha-CATH3 for fungal membranes and lack of toxicity to mammalian cells is promising for future development of a novel antifungal drug.

## Methods

Detailed methods are provided in the Supplementary information. Briefly, Tasmanian devil cathelicidins were mined from the *Sarcophilus harrisii* reference genome version 7.0 2011 on Ensembl using tammar wallaby and opossum cathelicidins as BLAST query sequences. Devil cathelicidins were aligned to other marsupial and eutherian cathelicidins using ClustalW^43^, and phylogenetic analysis was performed with nucleotide alignment using neighbour-joining method in MEGA5.2^44^.

Tissue expression of devil cathelicidin prepropeptides was determined by real-time PCR on a range of secretory, immune, reproductive, gastrointestinal and respiratory tissues, as well as BLAST searches of the milk transcriptome^31^. Opportunistic tissue samples for real-time PCR were collected from Taronga Zoo (Sydney) from devils which were subject to euthanasia due to old age and advanced disease. The mature peptides of all six devil cathelicidins were synthesised by ChinaPeptides Co. Ltd. and their antimicrobial activity against bacterial and fungal pathogens determined by broth microdilution susceptibility assay according to clinical laboratory standards institute guidelines. Antimicrobial activity was expressed as minimum inhibitory concentration (MIC). Cytotoxicity was assessed in the human cell line A549 and haemolytic activity determined against human blood. Statistical significance of peptide effect on cell survival in comparison to the untreated control was calculated using a one sample t-test. Pouch microbiome analysis was performed on swabs from the pouch of lactating and non-lactating devils to detect changes in microbe populations. Pouch swab collection was carried out in accordance with the Save the Tasmanian Devil Program and was approved by the Animal Ethics Committee of the University of Sydney (permit number 681).

## Conclusion

The Tasmanian devil has a broad repertoire of cathelicidins which likely evolved to protect their immunologically naive young. Their wide expression profile and broad-spectrum antimicrobial activity suggests devil cathelicidins are involved in epithelial defence in the pouch and at other sites on the body. In addition, devil cathelicidins may provide passive immunity to the young via the milk and could modulate immune responses to enhance protection during immune development in the pouch. Further research is required to characterise the immunomodulatory properties of devil cathelicidins.

## Additional Information

**How to cite this article**: Peel, E. *et al*. Cathelicidins in the Tasmanian devil (*Sarcophilus harrisii*). *Sci. Rep.* **6**, 35019; doi: 10.1038/srep35019 (2016).

## Supplementary Material

Supplementary Information

## Figures and Tables

**Table 1:** The amino acid sequence of six Tasmanian devil cathelicidins, their molecular weight, charge at pH7 and hydrophobic percentage.

Cathelicidin	Sequence	Molecular weight (g/mol)	Charge at pH7	Hydrophobic %
**Saha-CATH1**	GIKHILFMAKTKLPRATCTAEIKENCDRKK	3445.15	5.1	36.67
**Saha-CATH2**	TFKRKNGSRKNGHRPGGYSLIALGNKKVLKAPYMESI	4117.81	8.1	32.43
**Saha-CATH3**	KRMGIFHLFWAGLRKLGNLIKNKIQQGIENFLG	3841.61	5.1	45.45
**Saha-CATH4**	KREDFLDQIIRDFRNFIYQKYRRLRDEFRKLRDILSG	4820.52	3.9	32.43
**Saha-CATH5**	KRIGLIRLIGKILRGLRRLG	2301.93	6.9	45.00
**Saha-CATH6**	KRIRFFERIRDRLRDLGNRIKNRIRDFFS	3794.42	6.9	34.48

**Table 2:** Antimicrobial activity of Tasmanian devil cathelicidins Saha**-**CATH3, 5 and 6 against bacteria and fungi, expressed as the minimum inhibitory concentration (MIC).

Strain	MIC (μg/mL)			
	CATH3	CATH5	CATH6	Antibiotics
*E*. *coli* ATCC 25922	>64	32	>64	8^a^
*E*. *coli*	>64	32	>64	—
*S*. *aureus* ATCC 29213	>64	64	>64	0.125^b^
*S*. *aureus*	>64	64	>64	—
Methicillin resistant *S*. *aureus* (MRSA)	>64	32	>64	—
*P*. *aeruginosa* ATCC 27853	>64	>64	>64	8^b^
*P*. aeruginosa*	>64	64	>64	—
*E*. *faecalis* ATCC 29212	>64	>64	>64	1^a^
*E*. *faecalis*	>64	>64	>64	—
Vancomycin resistant *E*. *faecalis* (VREF)	>64	32	64	—
*S*. *pneumoniae* ATCC 49619	>64	64	64	0.125^a^
*S*. *pyogenes* ATCC 19615	>64	32	64	—
*S*. *agalactiae* ATCC 12386	>64	64	64	—
*S*. *agalactiae*	>64	64	>64	—
*S*. *mutans*	>64	>64	>64	—
*S*. *oralis/mitis* group	>64	64	64	—
*S*. *dysgalactiae* subs. e*quisimilis*	>64	>64	>64	—
*S*. *anginosus*	>64	32	64	—
*S*. *salivarius*	>64	>64	>64	—
*S*. *lutetiensis*	>64	>64	>64	—
*S*. equi*	>64	64	>64	—
*K*. pneumoniae*	>64	>64	>64	—
*N*. asteroides*	>64	>64	>64	—
*L*. monocytogenes*	>64	>64	>64	—
*P*. multocida*	>64	>64	>64	—
*C*. *parapsilosis* ATCC 22019	>64	>64	>64	2^c^
*C*. *krusei* ATCC 6258	>64	64	64	—
*C*. *albicans*	>64	>64	>64	—
*C*. *glabrata*	>64	>64	>64	—
*C*. *neoformans*	16	32	64	—
*C*. *gattii*	>64	64	64	—

Saha**-**CATH1, 2 and 4 did not show antimicrobial activity at the concentrations tested and hence are not included in the table. The MIC values for ^a^ampicillin, ^b^tetracycline and ^c^fluconazole fell within the acceptable limit for ATCC strains according to CLSI guidelines. *denotes animal isolate, otherwise all other bacteria are human clinical isolates or ATCC strains.
