# Supplementary material for: Cathelicidins in the Tasmanian devil (Sarcophilus harrisii)
Source: Sci Rep. 2016 Oct 11;6:35019. doi: 10.1038/srep35019 (PMC5057115; doi:10.1038/srep35019)
Supplement: Supplementary Information [file srep35019-s1.pdf]

Cathelicidins in the Tasmanian devil (*Sarcophilus harrisii*)

Peel, E, Cheng, Y, Djordjevic, JT, Fox, S, Sorrell, TC & Belov, K

Supplementary methods

Cathelicidin gene characterization

Tasmanian devil cathelicidins were identified using tammar wallaby and opossum cathelicidins as BLAST query sequences, which were aligned to the Sarcophilus harrisii reference genome version 7.0 2011 on Ensembl (available from http://www.ensembl.org/Sarcophilus_harrisii) with default parameters.

A multiple sequence alignment was constructed using ClustalW [1] in BioEdit [2] with coding sequences from human CAMP (NM_004345.4), mouse CAMP (NM_009921.2), sheep SMAP-29 (L46854.1), pig PMAP-37 (NM_001123149.1) & protegrin-1 (NM_213863.1), cow Bac5 (NM_174826.3), BMAP-27 (NM_174832.3) & BMAP-28 (NM_174510.3), chicken fowlicidin-1 (DQ092350.1), opossum Modo-CATH1 to 7 and 9 to 12 (all sequences from the immunome database for marsupials and monotremes (IDMM)[3]), tammar wallaby MaeuCath1 (EF624481.1), MaeuCath2 (157042605 IDMM), MaeuCath3 (EF624483.1), MaeuCath4 (157042609 IDMM), MaeuCath5 (157042610 IDMM), MaeuCath6 (EF624486.1), MaeuCath7 (EF624487.1) & MaeuCath8 (217038880 IDMM), and platypus Oran-CATH1 to 7 (all sequences from IDMM) cathelicidins. Signal peptide sequence was predicted using SignalP 4.1 [4]. Amino acid similarity and identity scores were calculated in BioEdit using the BLOSUM62 matrix. The sequence alignment was used to construct a Neighbour-Joining Phylogenetic tree in MEGA5.2[5] using p-distance, maximum composite likelihood and Jukes-Cantor model, pairwise deletion and 500 bootstrap replicates. All three models produced consistent results.

Expression profile

Relative expression quantification of six devil cathelicidin genes was analysed in 12 tissue types using real-time PCR. Opportunistic samples were collected from two female devils from the Taronga Zoo (Sydney) which were subject to euthanasia due to old age and advanced disease. A range of tissue samples were collected after euthanasia was performed, including whole blood, spleen, lymph node, oral mucosa, skin, pouch, ovary, uterus, liver, lung, small intestine, and heart. Blood was collected into RNAprotect Animal Blood Tubes (Qiagen) and other tissue samples were preserved in RNAlater (Sigma-Aldrich). Total RNA was extracted from blood using the RNeasy Protect Animal Blood Kit (Qiagen) and from stabilised tissues using the RNeasy Mini Kit (Qiagen). Quality of RNA was evaluated on a Bioanalyzer (Agilent Technologies). All samples had a RNA Integrity Number (RIN) greater than 7.5 except for one small intestine sample with RIN = 6.5 and a lung sample with RIN = 6.7. Approximately 100 ng of RNA was used for cDNA synthesis using the SuperScript VILO Master Mix (Invitrogen).

Real-time PCR primers for devil cathelicidin genes were designed using the software Oligo [6], with forward and reverse primers located on different exons and PCR amplicon size ranging between 92 and 146 bp (Table S1, see below). Two previously established reference genes, *GAPDH* [7] and *GUSB* [8], were used for qPCR data normalization. Real-time PCRs were carried out on a RotorGene 6000 in a total volume of 20 µl, containing 10 µl 2x Quantifast Sybr Green PCR Master Mix (Qiagen), 0.5 µM each of forward and reverse primers, and approximately 50 ng cDNA. All samples were analyzed in triplicates with no-template negative controls included for each gene in each run. PCR conditions were as follows: an initial enzyme activation step of 95 °C 5 minutes; 40 cycles of two-step cycling of 95 °C for 10 seconds and 60 °C for 30 seconds; a final heating step from 50 °C to 99 °C with

fluorescence signal collected every 1 °C to generate a melting curve. Standard curves were generated for all genes by making four 1:3 serial dilutions of a composite sample containing equal parts of analysed cDNA samples. R^2 of the standard curves were higher than 0.98 and PCR efficiencies ranged between 0.96 and 1.04 (Table S1). Data analysis was performed using Pfaffl method in software REST 2009 v2.0.13 [9]. Expression of target genes was normalized to the two reference genes taking into account different PCR efficiencies. For each cathelicidin gene, relative fold expression was calculated in comparison to the lowest expressing tissue. Additionally, BLAST searches were conducted against the milk transcriptome [10].

Pouch microbiome sequencing and analysis

Six pouch swabs, three from devils with three to four pouch young and three from non-lactating devils, were collected in Takone, Tasmania, in late June. Sampling was carried out by the Save the Tasmanian Devil Program during a routine monitoring trip and was approved by the Animal Ethics Committee of the University of Sydney (permit #681). Swabs were stored at -80°C until microbial DNA extraction was performed using the QIAamp UCP Pathogen Mini Kit (Qiagen). PCR amplicons of the bacterial 16S rRNA gene V3-V4 region (341F-806R) were generated and sequenced on an Illumina MiSeq system using paired 300-bp reads by the Australian Genome Research Facility (Brisbane). Sequence data was processed and analysed using the QIIME (v1.9) pipelines [11]. Paired-end reads were demultiplexed, joined, and quality-filtered using default parameters except for the following: minimum 100 bp of overlap with <20% differences required to join paired reads, and a maximum unacceptable Phred quality score of 29 in the joined sequences. Operational taxonomic units (OTUs), defined as groups of sequences with >97% similarity, were picked de novo with singletons removed. OTUs were assigned taxonomy with the UCLUST method [12] using the latest release of Greengenes (13_8) 0.97 OTUs dataset as reference. Alpha diversity, which reflects the bacterial phylotype richness in a sample, and beta diversity, which shows dissimilarity between samples, were calculated based on rarefied OTU tables with 40,000 sequences used for rarefaction. Lactating and nonlactating groups were compared in the alpha diversity and UniFrac distances (both weighted and unweighted) using the Monte Carlo method (999 permutations) to evaluate significance of differences. OTUs that showed significantly different frequencies between the two sample groups were identified by performing Wilcoxon rank sum tests.

Peptide synthesis

Mature peptide cleavage site of devil cathelicidins was predicted using ExPasy peptide cutter (http://web.expasy.org/peptide_cutter/) with neutrophil elastase. Molecular weight of each mature peptide and charge at pH 7 was calculated using Protein Calculator v3.4 (http://protcalc.sourceforge.net/, May 2013). Hydrophobic percentage was calculated using Peptide 2.0 peptide hydrophobicity/hydrophilicity analysis (http://peptide2.cpm/N_peptide_hydrophobicity_hydrophilicity.php, 2016) Mature peptides were synthesised, confirmed by mass spectrometry and purified to >95% by high performance liquid chromatography (HPLC) (ChinaPeptides Co. Ltd.)

Antimicrobial activity

Antimicrobial activity was determined using a broth microdilution susceptibility assay according to clinical laboratory standards institute (CLSI) guidelines in 96 well plates. Assay was performed in duplicate, on two separate occasions. Human and animal clinical isolates of bacteria and fungi, as well as American type culture collection (ATCC) strains were tested and are outlined in table 1. Cathelicidin stocks were prepared in DMSO, then serially diluted, twofold and in duplicate, in Mueller Hinton broth (MHB) with or without 10% lysed horse blood for bacteria, and yeast nitrogen

base (YNB) for fungi, from a concentration of 64µg/mL to 0.125µg/mL in a final volume of 100µL. Two antibiotics, ampicillin and tetracycline, were included as a positive control for bacteria, and the antifungal drug fluconazole for fungi. A well containing cells and media only was used as a "no drug" control to ensure that cultures grew normally. Bacteria were subcultured on Mueller Hinton agar (MHA) or Sheep blood agar (BA) for 24 hours at 35°C for *Staphylococcus* spp., Pseudomonas spp., Enterococcus spp. and E.coli, with 5% CO_2 for *Streptococcus* spp., *K.pneumoniae*, *P.multocida*, *L.monocytogenes* and *N.asteroides*. Similarly, fungi were subcultured on Sabouraud agar (SAB) at 35°C for 24 hours prior to the test. Colonies were resuspended in saline and the OD_{530} measured with a McFarland reader. If necessary the suspension was diluted to 0.45-0.55 McFarland units with saline. Following a 1:150 dilution for bacteria and twostep dilution for fungi (1:50, 1:20), 100 µL of microorganisms ($1x10^6$ – $5x10^5$ cells/mL) were pipetted into wells 2-12, the first well acting as a negative control. Colony counts were performed using a 1:1000 dilution for bacteria and 1:10 for fungi spread onto MHA/BA or SAB plates respectively. The 96 well microdilution and agar plates were incubated for 24 hours at 35°C (*Escherichia coli*, *Staphylococcus* spp., *Enterococcus* spp. and *Pseudomonas* spp.), with 5% CO_2 (*Streptococcus* spp. *Pasteurella multocida*, *Klebsiella pneumoniae*, *Listeria monocytogenes* and *Nocardia asteroids*) for bacteria, and 35°C for 48 hours (*Candida* spp.) or 72 hours (*Cryptococcus* spp.) for fungi. Assays Antimicrobial activity was expressed as minimum inhibitory concentration (MIC), which was defined as the lowest concentration of cathelicidin preventing visible growth, relative to the negative control. Colonies which grew on agar plates were also counted.

Cytotoxicity

Cytotoxicity was assessed in the human A549 cell line maintained in Dulbecco's Modification of Eagle's Medium (DMEM) supplemented with 10% foetal bovine serum (FBS), 2mM L-glutamine, 50units/mL penicillin and 50µg/mL streptomycin. Cells were passaged every four days. For the assay, cells were seeded at 1 x 10^5 cells/mL and 100µL transferred to a 96 well plate, omitting three wells for the negative control which contained DMEM only. The plate was incubated for 18 hours at 37°C 5% CO_2 95% humidity. Cathelicidin stocks were prepared at 1mg/mL in DMEM and serial dilution performed in duplicate. Cathelicidin dilutions were transferred to the seeded 96 well plates, resulting in a final peptide concentration of 500µg/mL to 1.9µg/mL. An untreated growth control of DMEM and positive control of dimethylsulfoxide (DMSO) (1%) were also included. Following incubation for a further 24 hours, cell viability was determined using alamar blue (Invitrogen) which was added to each well. The plates were incubated for 24 hours, then the absorption measured at 570nm and 620nm using the Victor 3 multilabel plate reader (PerkinElmer). The assay was performed on two separate occasions. Toxicity of cathelicidins was calculated according to Invitrogen recommendations and expressed as percentage cell survival compared to the untreated growth control. A significant difference between cathelicidin treated and control cell survival was tested using a one sample t-test (p<0.05).

Haemolytic activity

Haemolytic activity was assessed using 10mL of human blood collected into potassium-EDTA vacutainers (BD). The blood was washed three times with 30mL of calcium and magnesium-free Dulbecco's phosphate buffered saline (DPBS) and cells were collected by centrifugation at 2000 x g for 10 minutes. Cells were resuspended in 20mL of DPBS and stored at 4°C for up to two weeks. Cathelicidin solutions were prepared at 1mg/mL in DPBS and serial dilution performed in duplicate with two replicates. Then 100uL of cell suspension was mixed with 100uL of cathelicidin dilution, giving a final cathelicidin concentration of 500mg/mL to 0.9mg/mL. A negative control of DPBS and

positive control of Triton X-100 (1%) were also included. The mixtures were incubated at 37°C for one hour then centrifuged at 2000 x g for 3 minutes. The supernatant was diluted one in ten with DPBS, then the absorption read at 544nm using Victor 3 multilabel plate reader (PerkinElmer). Percentage red blood cell lysis was calculated based on the value of 0% and 100% lysis determined by the absorption of less than PBS or more than 1% Triton X-100 respectively. In between 0 and 100% lysis was calculated using the following formula.

$$Lysis\ (\%) = A\ \times (absorbance\ of\ sample\ ) +\ B$$

Where:

$$A = \frac{Absorbance\ of\ Triton\ X - Absorbance\ of\ PBS}{100}$$

$$B = 0 - A\ \times Absorbance\ of\ PBS$$

A significant difference between the absorbance values of cathelicidin treated red blood cells and the negative control was tested using a one sample t-test ($p<0.05$).

Supplementary figures

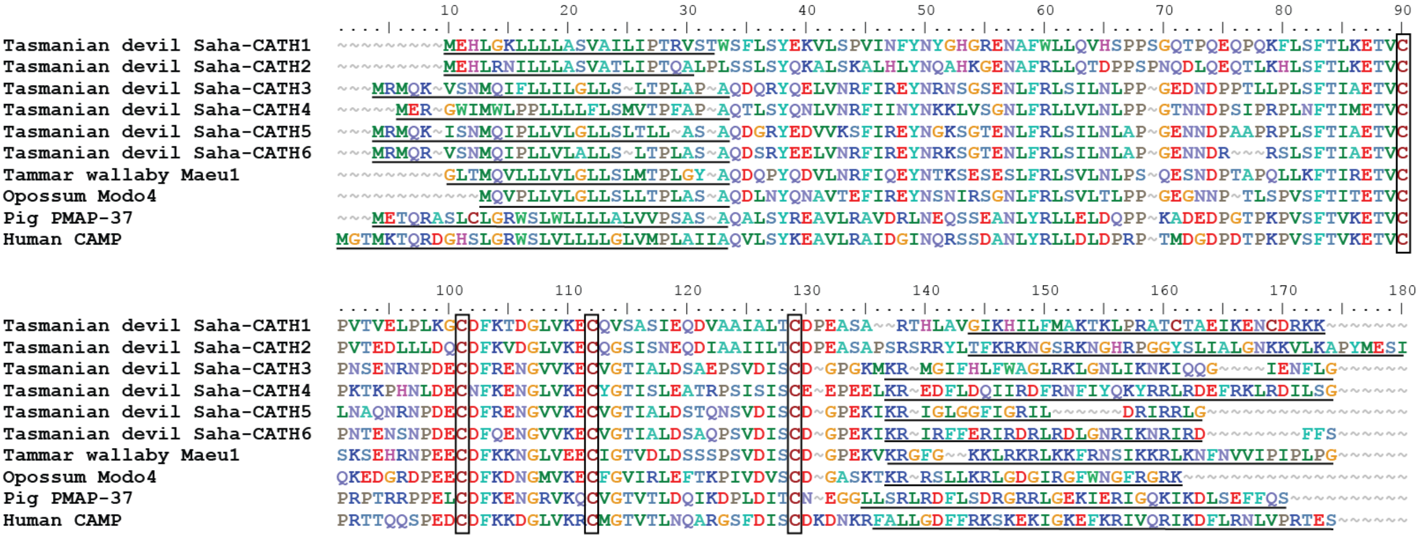

Supplementary figure S1. Multiple sequence alignment of Tasmanian devil cathelicidins Saha-CATH1 to 6, with tammar wallaby MaeuCath1, opossum Modo-CATH4, Human CAMP and Pig PMAP-37. Conserved cysteines within the cathelin domain are boxed and the predicted signal peptide and mature peptide is underlined.

Supplementary table S1. Tasmanian devil cathelicidin real-time PCR primers and efficiency.

Gene	Primers (5'–3')	Amplicon size (bp)	Standard curve R^2	PCR efficiency
Saha-CATH1	F: CTATGGACATGGGAGAGAGAATG<br>R: GAAACTTCTGGGGTTGCTCTTG	92	0.980	1.01
Saha-CATH2	F: TCATCTCTACAACCAAGCACACA<br>R: GTTTGTTCTTGAAGGTCCTGATTG	93	0.996	1.04
Saha-CATH3	F: CTGCTCAGGACCAAAGATACCA<br>R: CAAAGGTAAAAGAGTAGGAGGATCA	146	0.981	0.97
Saha-CATH4	F: CCAAGACCAAACCACACAATCTG<br>R: TCGTGTAGCTTCCAAGGAGATG	95	0.992	1.00
Saha-CATH5	F: GTGTGCCTCAATGCTCAAAATC<br>R: ATATCAACGGAGTTCTGGGTAGA	116	0.991	0.96
Saha-CATH6	F: GAATACAACAGGAAGTCAGGAACT<br>R: CTCAGAGATCGACGATCATTG	92	0.995	1.00
GAPDH*	F: ATATGATTCCACCCATGGCAAGTTCAA<br>R: GACTCAACCACGTATTCGGCTC	150	0.998	0.99
GUSB*	F: CTGCTGCCTATTATTTCAAGAC<br>R: CAAGATCCAATTCAGGCTTAG	102	0.994	1.02

*GAPDH and GUSB primers were designed by Murchison, et al. [7] and Morris and Belov [8], respectively.

References

1 Thompson, J. D., Higgins, D. G. & Gibson, T. J. Clustal W: improving the sensitivity of progressive multiple sequence alignment through sequence weighting, position specific gap penalties and weight matrix choice. *Nucleic Acids Research* **22**, 4673-4680 (1994).

2 BioEdit v. v7.2.2 (Ibis Biosciences, 2013).

3 Wong, E., Papenfuss, A. & Belov, K. Immunome database for marsupials and monotremes. *BMC Immunology* **12** (2011).

4 Peterson, T. N., Brunak, S., von Heijne, G. & Nielsen, H. SignalP4.0: discriminating signal peptides from transmembrane regions. *Nature Methods* **8**, 785-786 (2011).

5 Tamura, K. *et al.* MEGA5: Molecular Evolutionary Genetic Analysis using Maximum likelihood, Evolutionary distance and Maximum parsimony methods. *Molecular Biology and Evolution* **28**, 2731-2739 (2011).

6 Rychlik, W. in *PCR Primer Design* (ed Anton Yuryev) 35-59 (Humana Press, 2007).

7 Murchison, E. P. *et al.* The Tasmanian Devil Transcriptome Reveals Schwann Cell Origins of a Clonally Transmissible Cancer. *Science* **327**, 84-87, doi:10.1126/science.1180616 (2010).

8 Morris, K. & Belov, K. Does the devil facial tumour produce immunosuppressive cytokines as an immune evasion strategy? *Veterinary Immunology and Immunopathology* **153**, 159-164, doi:http://dx.doi.org/10.1016/j.vetimm.2013.02.008 (2013).

9 Pfaffl, M. W., Horgan, G. W. & Dempfle, L. Relative expression software tool (REST©) for group-wise comparison and statistical analysis of relative expression results in real-time PCR. *Nucleic Acids Research* **30**, e36, doi:10.1093/nar/30.9.e36 (2002).

10 Hewavisenti, R. V. *et al.* The identification of immune genes in the milk transcriptome of the Tasmanian devil (*Sarcophilus harrisii*). *PeerJ* **4** (2016).

11 Caporaso, J. D. *et al.* QIIME allows analysis of high-throughput community sequencing data. *Nature Methods* **7**, 335-336 (2010).

12 Edgar, R. C. Search and clustering orders of magnitude faster than BLAST. *Bioinformatics* **26**, 2460-2461 (2010).
